# Phase Ib Study of the Histone Deacetylase 6 Inhibitor Citarinostat in Combination With Paclitaxel in Patients With Advanced Solid Tumors

**DOI:** 10.3389/fonc.2021.786120

**Published:** 2022-01-07

**Authors:** Michael S. Gordon, Geoffrey I. Shapiro, John Sarantopoulos, Dejan Juric, Brian Lu, Angeliki Zarotiadou, Jamie N. Connarn, Yvan Le Bruchec, Calin Dan Dumitru, R. Donald Harvey

**Affiliations:** ^1^ Departments of Hematology and Medical Oncology, HonorHealth Research Institute, Scottsdale, AZ, United States; ^2^ Department of Medical Oncology, Dana-Farber Cancer Institute, Brigham and Women’s Hospital and Harvard Medical School, Boston, MA, United States; ^3^ Department of Medicine, Division of Medical Oncology & Hematology, Institute for Drug Development, Mays Cancer Center at UT Health San Antonio MD Anderson Cancer Center, San Antonio, TX, United States; ^4^ Massachusetts General Hospital Cancer Center, Boston, MA, United States; ^5^ Department of Medicine, Harvard Medical School, Boston, MA, United States; ^6^ Bristol Myers Squibb, Princeton, NJ, United States; ^7^ Celgene Research S.L.U., a Bristol-Myers Squibb Company, Boudry, Switzerland; ^8^ Department of Hematology and Medical Oncology, Winship Cancer Institute of Emory University and Emory University School of Medicine, Atlanta, GA, United States

**Keywords:** advanced solid tumors, HDAC inhibition, epigenetics, paclitaxel, combination therapy, citarinostat, histone acetylation, histone deacetylase

## Abstract

**Background:**

Citarinostat (CC-96241; previously ACY-241), an oral inhibitor of histone deacetylases (HDACs) with selectivity for HDAC6, has demonstrated synergistic anticancer activity with paclitaxel in multiple solid tumor models. Combination therapy using citarinostat with paclitaxel was evaluated in this phase Ib 3 + 3 dose-escalation study in patients with advanced solid tumors.

**Methods:**

Patients with previously treated advanced solid tumors received citarinostat 180, 360, or 480 mg once daily on days 1 to 21 plus paclitaxel 80 mg/m^2^ on days 1, 8, and 15 of 28-day cycles until disease progression or unacceptable toxicity. The primary endpoint was determination of the maximum tolerated dose (MTD). Secondary endpoints included safety, antitumor activity, pharmacokinetics, and pharmacodynamics.

**Results:**

Twenty patients were enrolled and received study treatment; 15 had received prior taxane therapy. No dose-limiting toxicities were reported at any dose; therefore, the MTD was not identified. Citarinostat 360 vs 480 mg was associated with reduced incidence and severity of neutropenia. Three patients experienced a confirmed partial response and 13 achieved stable disease. Pharmacokinetic parameters were linear up to citarinostat 360 mg, the dose at which the highest levels of histone and tubulin acetylation were observed in peripheral blood mononuclear cells.

**Conclusions:**

The combination of citarinostat plus paclitaxel showed an acceptable safety profile, with no unexpected or dose-limiting toxicities and potential evidence of antitumor activity in patients with heavily pretreated advanced solid tumors. Citarinostat 360 mg once daily is considered the recommended phase II dose for use in combination with paclitaxel 80 mg/m^2^ every 3 of 4 weeks. This trial is registered on ClinicalTrials.gov (NCT02551185).

## Introduction

Histone deacetylases (HDACs) are commonly overexpressed in many types of cancers (1, 2), an observation that has led to the emergence of cancer therapies targeting HDACs (3). HDAC inhibitors (HDACi) have been shown to reestablish normal histone acetylation patterns and demonstrate antitumorigenic effects, including cell death, cell cycle arrest, suppression of angiogenesis, and immunomodulation (1–6). However, monotherapy with pan-HDACi has demonstrated limited clinical benefit (7–9). Furthermore, development of pan-HDACi has been limited by high-grade toxicities, as reported in the PANORAMA1 study in myeloma (10).

Citarinostat (CC-96241; previously ACY-241) is an oral selective HDAC6 inhibitor that exhibits potent biochemical inhibition of HDAC6 with 13- to 18-fold reduced potency against the nuclear class I HDACs, including HDAC1, HDAC2, and HDAC3 (11). In a preclinical study using A2780 ovarian cancer cells, treatment with citarinostat (300 nM) for 24 hours increased α-tubulin acetylation, which is consistent with tubulin deacetylase HDAC6 inhibition. In addition, acetylation of histones (targeted by class I HDACs) was also increased but only at citarinostat doses exceeding 1 μM. Collectively, these preclinical data suggest that low-dose citarinostat may selectively inhibit HDAC6; however, increased citarinostat dosing may also inhibit class I HDAC isozymes.

HDAC6 inhibition may increase microtubule stability and has been shown to mechanistically enhance the tubule-stabilizing action of taxane chemotherapy, leading to increased cell death in preclinical models (12, 13). For example, pretreatment of anaplastic thyroid carcinoma cells with valproic acid, which inhibits HDAC6, improved the anticancer activity of paclitaxel (13). Similarly, class I HDAC inhibition may also augment the antitumor activity of taxanes; romidepsin, an RPD3-like class I HDACi, was shown to have a synergistic effect when combined with paclitaxel in a preclinical model of breast cancer (12, 14). In a phase II trial of patients with previously untreated stage IIIB or IV non-small cell lung cancer, vorinostat (a class I and II HDACi) plus carboplatin and paclitaxel resulted in a significantly higher response rate as well as a trend in improved progression-free survival and overall survival compared with carboplatin plus paclitaxel alone (15). However, the rate of grade 4 thrombocytopenia was notably higher with vorinostat plus chemotherapy vs chemotherapy alone. In a phase 1a/b first-in-human study in patients with relapsed or refractory multiple myeloma, citarinostat alone or in combination with pomalidomide and dexamethasone demonstrated promising safety and tolerability (16).

A preclinical study demonstrated that citarinostat (or the structurally related ricolinostat, the liquid formulation of citarinostat) in combination with paclitaxel suppressed the growth of multiple solid tumor lineages, including pancreatic, ovarian, and breast cancer cell lines, and significantly reduced the tumor volume in ovarian and pancreatic xenograft models compared with either agent alone (11). These results prompted us to conduct a phase Ib study designed to determine the safety, pharmacokinetics, pharmacodynamics, and preliminary antitumor activity of citarinostat plus paclitaxel in patients previously treated with standard regimens for advanced solid tumors.

## Materials and Methods

This study was approved by the institutional review board at each participating site. Study monitoring was conducted in compliance with the International Council for Harmonisation of Technical Requirements for Pharmaceuticals for Human Use and current Good Clinical Practice guidelines and the general ethical principles of the Declaration of Helsinki. Written informed consent was obtained from all patients before study entry. This trial is registered on ClinicalTrials.gov (NCT02551185).

### Patients

Patients aged ≥ 18 years with an incurable, histologically confirmed, metastatic or locally advanced solid tumor (defined as nonhematologic), evaluable by Response Evaluation Criteria in Solid Tumors (RECIST) version 1.1 and for which paclitaxel monotherapy was clinically appropriate, were included. Eligible patients must have received and experienced disease progression with standard treatment for the malignancy and had an Eastern Cooperative Oncology Group performance status (ECOG PS) of 0 to 2. Patients for whom no standard treatment was available were also included. Tumor stage at baseline was measured by the American Joint Committee on Cancer TNM or International Federation of Gynecology and Obstetrics (FIGO) staging system. Patients previously treated with HDACi were excluded.

### Study Design

**Figure 1:**
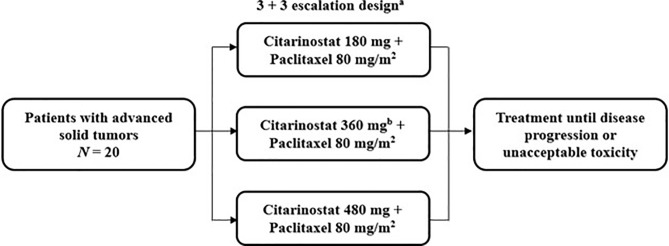
Study design of the phase Ib trial of citarinostat plus paclitaxel in patients with advanced solid tumors. ^a^28-day cycles with citarinostat administered orally once daily on days 1 to 21 at all doses and paclitaxel administered intravenously on days 1, 8, and 15. ^b^An additional 360-mg cohort was allowed if the maximum tolerated dose was not reached.

This study was a phase Ib, multicenter, single-arm, open-label, dose-escalation trial to determine the maximum tolerated dose (MTD) and evaluate the safety and preliminary antitumor activity of citarinostat in combination with paclitaxel in patients with advanced solid tumors (**Figure 1**). Patients were treated at 5 institutions in the United States.

Patients received citarinostat 180, 360, or 480 mg orally once daily on days 1 to 21 of 28-day treatment cycles, in a 3 + 3 dose-escalation design, in combination with paclitaxel 80 mg/m^2^ (as recommended by the prescribing information) (17) administered intravenously on days 1, 8, and 15 of each 28-day treatment cycle (the first 3 of 4 weeks [qw 3/4]); ≥ 3 patients were enrolled in each cohort. If 1 of 3 patients experienced a dose-limiting toxicity (DLT), the cohort was expanded to ≥ 6 patients. If ≤ 1 of 6 patients experienced a DLT, escalation continued to the next cohort. Dose escalation was continued successively to new cohorts of 3 patients each until the first DLT was observed, after which up to 3 additional patients would be treated at that dose level. The MTD was defined as the highest dose level at which < 2 of 6 patients experienced a DLT in a given dose cohort. Cohorts with additional citarinostat dose levels were allowed if the MTD was not identified. Patients who experienced a DLT or other unacceptable toxicity in cycle 1 were removed from study treatment. Patients were treated until disease progression or an unacceptable toxicity was experienced. The primary results of this study are reported here. Data cutoffs were October 4, 2019, for safety and efficacy results and November 27, 2017, for pharmacokinetic and pharmacodynamic analyses.

### Endpoints and Assessments

The primary objectives of the study were to assess DLTs and the MTD (if reached) and determine the recommended phase II dose (RP2D) and schedule of citarinostat in combination with paclitaxel. The primary endpoint of this study was to establish the MTD of citarinostat in combination with weekly paclitaxel. The secondary endpoints were antitumor activity as measured by the change from baseline in tumor measurements as documented by objective response, objective response rate (complete response + partial response [PR]) assessed according to RECIST 1.1, duration of response, disease control rate (complete response + PR + stable disease), and safety and tolerability (as measured by the proportion of patients experiencing any adverse event [AE], serious AE, AE leading to study drug discontinuation, DLTs, or incidence of specific AEs [including peripheral neurotoxicity]). AEs were coded using the Medical Dictionary for Regulatory Activities version 18.0, while severity was assessed by the investigator according to National Cancer Institute Common Terminology Criteria for Adverse Events version 4.03. Definitions of DLTs classified as related to citarinostat are outlined in **Table 1**. Other secondary endpoints included pharmacokinetics of the combination therapy and pharmacodynamics of citarinostat monotherapy.

**Table 1:** DLT definitions.

DLTs classified as related to citarinostat
Hematologic toxicity
• Grade 4 neutropenia (ANC < 500/μL) lasting > 7 days. If neutropenia is attributed to citarinostat, ANC is to be reassessed on day 6 to determine whether the AE is a DLT • Febrile neutropenia (ANC < 1000/μL with a single temperature > 38.3°C or 38.0°C for > 1 hour) • Grade 4 thrombocytopenia (platelet count < 25,000/μL) • Grade 3 thrombocytopenia (platelet count 25,000–50,000/μL) with clinically important bleeding • Grade 4 anemia (life-threatening consequences; urgent intervention indicated)
Nonhematologic toxicity
• All other grade 3/4 nonhematologic toxicities, with the following considerations: ◦ For grade ≥ 3 nausea, vomiting, and/or diarrhea to be considered a DLT, the event must have persisted for > 72 hours despite maximal medical intervention ◦ Transaminitis (AST and ALT) > 2 × increase over baseline lasting > 72 hours ◦ Delay of patient continuing to cycle 2 by > 7 days due to AEs related to citarinostat

AE, adverse event; ALT, alanine aminotransferase; ANC, absolute neutrophil count; AST, aspartate aminotransferase; DLT, dose-limiting toxicity.

Objective tumor response was assessed by caliper measurements or computed tomography/magnetic resonance imaging according to RECIST 1.1.

### Pharmacokinetics and Pharmacodynamics

Pharmacokinetic assessments of citarinostat and paclitaxel included maximum concentration (C_max_), time to C_max_ (T_max_), area under the curve (AUC) from 0 to 24 hours (AUC_0-24h_), AUC from 0 to infinity (AUC_0-∞_), and serum half-life (t_1/2_). Blood samples for pharmacokinetic assessments were collected on days 1 and 15 of cycle 1 before paclitaxel infusion (predose); immediately after paclitaxel infusion but before citarinostat dosing; and at 0.5, 1, 2, 4, 6, and 24 hours (on days 2 and 16) after citarinostat dosing.

Peripheral blood mononuclear cell (PBMC) samples were collected for pharmacodynamic evaluation of acetylated histones and tubulin. PBMCs were isolated by centrifugation using Ficoll-Paque PLUS (GE Healthcare 17-1440-02) and stored in 10% dimethyl sulfoxide freezing medium at −80°C until analysis. Levels of acetylated histone 2B lysine 5 (Ac-histone) and acetylated α-tubulin (Ac-tubulin), used as surrogate markers of HDAC6-specific inhibition, were assessed by flow cytometry in the CD3^+^ subset of PBMCs. Monoclonal mouse antihuman acetylated alpha tubulin (clone 6-11B-1; Sigma Aldrich T7451) and polyclonal rabbit antihuman acetylated histone 2B lysine 5 antibodies (Cell Signaling Technology 2574) were used, with appropriate fluorescent (DyLight 488) secondary antibodies (Kirkegaard & Perry Labs 072-03-18-06 and 072-03-15-16). For each patient, the Ac-tubulin or Ac-histone mean fluorescence intensities of the postdose samples were divided by the Ac-tubulin or Ac-histone predose mean fluorescence intensities after background subtraction. Pharmacodynamic analyses were performed for histone and tubulin acetylation samples obtained on days 1 and 15 of cycle 1 before paclitaxel infusion (predose); immediately after paclitaxel infusion but before citarinostat dosing; and at 0.5, 1, 2, 4, 6, and 24 hours (on days 2 and 16) after citarinostat dosing.

### Statistical Analyses

Patients were enrolled in this single-arm, open-label study using a 3 + 3 design. Up to 41 evaluable patients were planned to be enrolled based on the assumption that 24 evaluable patients would be enrolled in a total of 3 dose cohorts plus 1 intermediate dose cohort in the dose-escalation part of the study. This approach would have included 6 patients at the MTD and 17 evaluable patients in the dose-expansion part of the study. However, the expansion phase was not performed because the study was discontinued early, owing to a decision by the sponsor that was not related to patient safety issues.

Patients who received ≥ 1 dose of study drug were considered evaluable for safety. All patients who provided consent and met study eligibility criteria were considered to be the intent-to-treat population. All patients who received ≥ 80% of the target doses during cycle 1 were to be DLT-evaluable. Statistical analyses were primarily descriptive, as the goals were to evaluate DLTs and the MTD and to determine the RP2D and schedule of citarinostat in combination with paclitaxel. Continuous variables were summarized using descriptive statistics (number, mean, standard deviation, median, minimum, and maximum), while categorical variables were summarized as the number and percentage within each classification.

Pharmacokinetic parameters were analyzed based on a noncompartmental model approach (Phoenix WinNonlin; Certara). Pharmacodynamic analyses were expressed as the fold change in the levels of Ac-histone and Ac-tubulin between pre- and postdose time points for each patient.

## Results

### Patient Disposition and Treatment Duration

A total of 20 patients were enrolled and treated. The citarinostat dose was escalated (180, 360, or 480 mg) in combination with paclitaxel 80 mg/m^2^. Six of these patients (30%) received citarinostat 360 mg as part of an additional cohort to further evaluate the tolerability of the combination of citarinostat and paclitaxel and to confirm the RP2D in those with disease that had progressed following taxane therapy. Among all patients, treatment discontinuations were due to progressive disease (PD; 80%), AEs (10%), physician decision (5%), and patient withdrawal (5%). The median treatment duration for all patients was 13.5 weeks, with a median of 4 cycles started (range, 1-26 cycles). Two deaths occurred due to clinical progression during follow-up (both in patients with ovarian tumors).

### Baseline Characteristics

Patient demographics and baseline characteristics are described in **Table 2**. The median age was 60 years, and most patients were female (65%) and had an ECOG PS of 1 (70%). The most common tumor types were ovarian (*n* = 6) and breast (*n* = 4), and the most common tumor stages at baseline were TNM IV (*n* = 14) and FIGO IVA (*n* = 2). A total of 15 patients who had previously been treated with taxanes were enrolled across all cohorts.

**Table 2:** Patient demographics and baseline characteristics: safety population.

Variable	Citarinostat 180 mg	Citarinostat 360 mg	Citarinostat 480 mg
	*n* = 3	*n* = 11	*n* = 6
Age, median, years	57	60	59
< 65 years, n (%)	3 (100)	6 (55)	3 (50)
≥ 65 years, n (%)	0	5 (45)	3 (50)
Sex, n (%)			
Female	3 (100)	6 (55)	4 (67)
Male	0	5 (45)	2 (33)
Race, n (%)			
White	3 (100)	10 (91)	4 (67)
Black or African American	0	1 (9)	2 (33)
ECOG PS, n (%)			
0	1 (33)	3 (27)	1 (17)
1	2 (67)	7 (64)	5 (83)
2	0	1 (9)	0
Primary cancer type, n (%)			
Ovarian	3 (100)	3 (27)	0
Breast	0	2 (18)	2 (33)
Cervical	0	1 (9)	1 (17)
Head and neck	0	1 (9)	1 (17)
Gastric/GE junction	0	1 (9)	1 (17)
Lung	0	1 (9)	0
Prostate	0	1 (9)	0
Mesothelioma	0	1 (9)	0
Biliary tract	0	0	1 (17)
Disease stage at baseline, n (%)			
TNM IIIB	0	0	1 (17)
TNM IV	1 (33)	8 (73)	5 (83)
FIGO IIB	1 (33)	0	0
FIGO IVA	1 (33)	1 (9)	0
FIGO IVB	0	1 (9)	0

ECOG PS, Eastern Cooperative Oncology Group performance status; FIGO, International Federation of Gynecology and Obstetrics; GE, gastroesophageal.

### Safety

No DLTs were observed; therefore, the MTD was not reached. All 20 patients experienced ≥ 1 treatment-emergent AE (TEAE), and 8 (40%) experienced a serious TEAE; 14 patients (70%) experienced ≥ 1 grade 3/4 TEAE (**Table 3**). The most common (≥ 10% incidence) grade 3/4 TEAEs at all doses were peripheral neuropathy (25%) and anemia (15%), followed by dehydration, hypophosphatemia, neutropenia, and urinary tract infection (10% each). Overall, a lower incidence and reduced severity of neutropenia were observed at the 360-mg dose (*n* = 1; grade 1) vs the 480-mg dose (*n* = 3; 1 each of grades 2, 3, and 4). One serious TEAE related to treatment (infected fistula) was reported in a patient treated with citarinostat 360 mg. No grade 5 TEAEs were reported.

**Table 3:** Incidence of the most common any-grade and grade 3/4 TEAEs (all doses): safety population.

TEAE, *n* (%)	*N* = 20
Any grade (≥ 20%)	
**Patients with any TEAE**	**20 (100)**
Peripheral neuropathy^a^	11 (55)
Anemia	10 (50)
Hypomagnesemia	9 (45)
Alopecia	8 (40)
Fatigue	7 (35)
Decreased appetite	7 (35)
Diarrhea	6 (30)
Hyperglycemia	6 (30)
Nausea	6 (30)
Vomiting	6 (30)
Pyrexia	5 (25)
Hypokalemia	5 (25)
Dehydration	5 (25)
Dysgeusia	4 (20)
Hypophosphatemia	4 (20)
Leukopenia	4 (20)
Neutropenia	4 (20)
Grade 3/4 (≥ 10%)	
**Patients with any grade 3/4 TEAE**	**14 (70)**
Peripheral neuropathy^b^	5 (25)
Anemia	3 (15)
Dehydration	2 (10)
Hypophosphatemia	2 (10)
Neutropenia	2 (10)
Urinary tract infection	2 (10)

TEAE, treatment-emergent adverse event.

a Reported as a grouped term (peripheral sensory neuropathy, neuropathy peripheral, paresthesia, muscular weakness, and gait disturbance).

b Reported as a grouped term (peripheral sensory neuropathy, neuropathy peripheral, and paresthesia).

Bold identifies the total number of patients in each of those groups (any-grade TEAEs and grade 3/4 TEAEs).

### Response

Nineteen of 20 patients were evaluable for response. At the 480-mg dose, 1 confirmed PR was observed. Two confirmed PRs were observed at the 360-mg dose. The 3 patients who experienced a PR all previously had disease progression with taxane-based treatment. The PRs occurred in patients with head and neck, breast, and lung cancers (*n* = 1 each). A best response of stable disease was achieved in 3 patients at 180 mg, 6 at 360 mg, and 4 at 480 mg; 9 of these patients experienced progression with prior taxane treatment. Stable disease was achieved in patients with ovarian (*n* = 4), gastric/gastroesophageal junction (*n* = 2), cervical (*n* = 2), and head and neck, breast, biliary tract, prostate, and lung (*n* = 1 each) tumors. One of the patients with ovarian cancer who had received a taxane as immediate prior treatment with progressive disease as the best response was treated at 360 mg and experienced prolonged stable disease over 7 cycles.

Overall, 3 patients experienced PD: 2 at 360 mg and 1 at 480 mg. Across cohorts, PD was experienced by 2 patients who previously had PD with taxane treatment. Tumor types in patients experiencing PD were breast (*n* = 2) and ovarian (*n* = 1).

**Figure 2:**
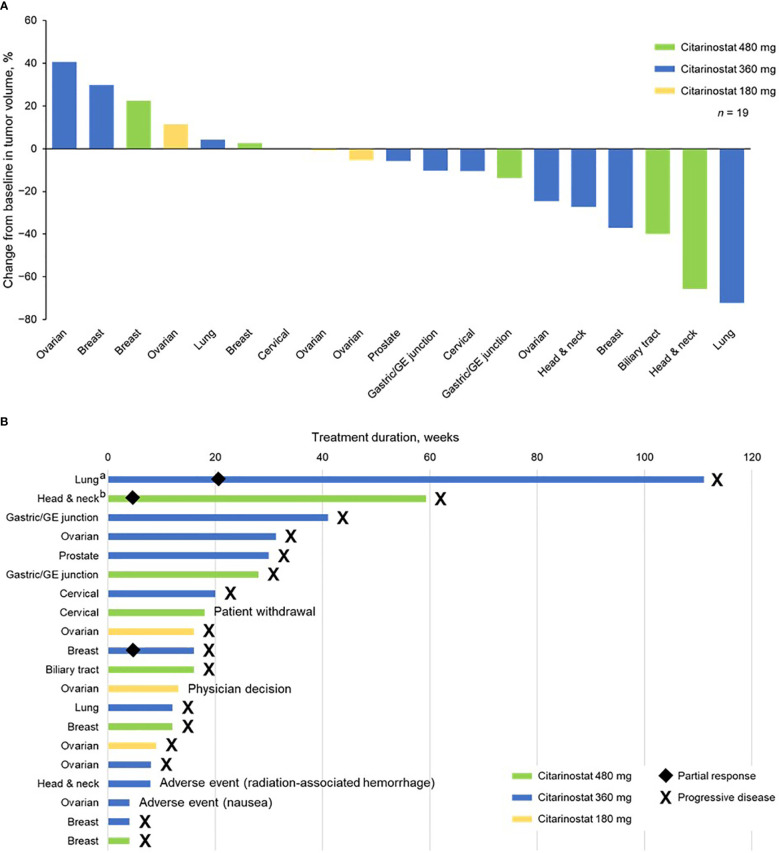
**(A)** Tumor shrinkage by enrolled population (1 patient with ovarian cancer did not have a postbaseline efficacy assessment and was not included in this analysis) and **(B)** treatment duration by individual patients (safety population). ^a^Received citarinostat as monotherapy beginning in cycle 25. ^b^Received citarinostat as monotherapy beginning in cycle 11 (paclitaxel withdrawn due to grade 3 neuropathy). GE, gastroesophageal.

Tumor shrinkage and treatment duration data are shown in **Figure 2**. Twelve patients (63.2%) experienced tumor shrinkage.

### Pharmacokinetics/Pharmacodynamics

Citarinostat exposure (AUC and C_max_) nearly doubled from the 180- to 360-mg dose but appeared to be saturated above 360 mg, with a small increase observed in citarinostat exposure from 360 to 480 mg (**Table 4**). Generally, T_max_ and t_1/2_ were similar, regardless of dose or following multiple doses (cycle 1 day 1 vs day 15). With citarinostat 360 and 480 mg, citarinostat exposure was consistent from cycle 1 day 1 to day 15. Paclitaxel exposure decreased with increasing dose levels of citarinostat from 180 to 480 mg. Paclitaxel t_1/2_ was similar regardless of dose or following multiple doses (cycle 1 day 1 vs day 15).

**Table 4:** Citarinostat and paclitaxel pharmacokinetics at different citarinostat doses.^a^

Parameter	Citarinostat dose, cycle 1 day 1			Citarinostat dose, cycle 1 day 15		
	180 mg	360 mg	480 mg	180 mg	360 mg	480 mg
	*n* = 3	*n* = 10	*n* = 6	*n* = 3	*n* = 10	*n* = 5^b^
Pharmacokinetics for citarinostat						
C_max_, mean (SD), ng/mL	1614 (851)	3047 (1644)	3235 (1138)	3067 (1980)	3807 (2867)	2998 (1501)
T_max_, median (min–max), h	2.00 (2.00–2.00)	1.50 (1.00–4.00)	1.50 (1.00–4.00)	1.00 (1.00–1.00)	1.00 (1.00–6.00)	1.00 (1.00–4.00)
t_1/2_, mean (SD), h	2.8 (0.11)	2.9 (0.36)	2.8 (0.26)^c^	2.9 (0.11)	3.3 (0.44)^d^	3.4 (0.72)
AUC_0-24h_, mean (SD), h•ng/mL	5812 (2082)	11,622 (3458)	13,248 (1324)	7715 (3738)	11,526 (4661)	13,314 (2691)
AUC_0-∞_, mean (SD), h•ng/mL	5820 (2083)	11,650 (3464)	13,575 (1220)^c^	7725 (3741)	12,682 (3266)^d^	13,430 (2711)
Pharmacokinetics for paclitaxel						
C_max_, mean (SD), ng/mL	4820 (2050)	2988 (627)	2355 (578)	5433 (2331)	3597 (1199)	2820 (1172)
t_1/2_, mean (SD), h	9.0 (0.93)	9.5 (1.3)	10.8 (2.2)	9.5 (1.6)	9.9 (2.3)	9.8 (4.4)
AUC_0-24h_, mean (SD), h•ng/mL	5986 (1284)	3837 (594)	3260 (705)	7537 (3677)	4985 (1272)	3183 (1044)
AUC_0-∞_, mean (SD), h•ng/mL	6612 (1363)	4354 (696)	3845 (867)	8446 (3744)	5692 (1417)	3531 (1441)

AUC, area under the curve; C_max_, maximum concentration; max, maximum; min, minimum; T_max_, time to maximum concentration.

a Data cutoff: November 27, 2017.

b n = 4 for paclitaxel pharmacokinetics.

c n = 5.

d n = 9.

**Figure 3:**
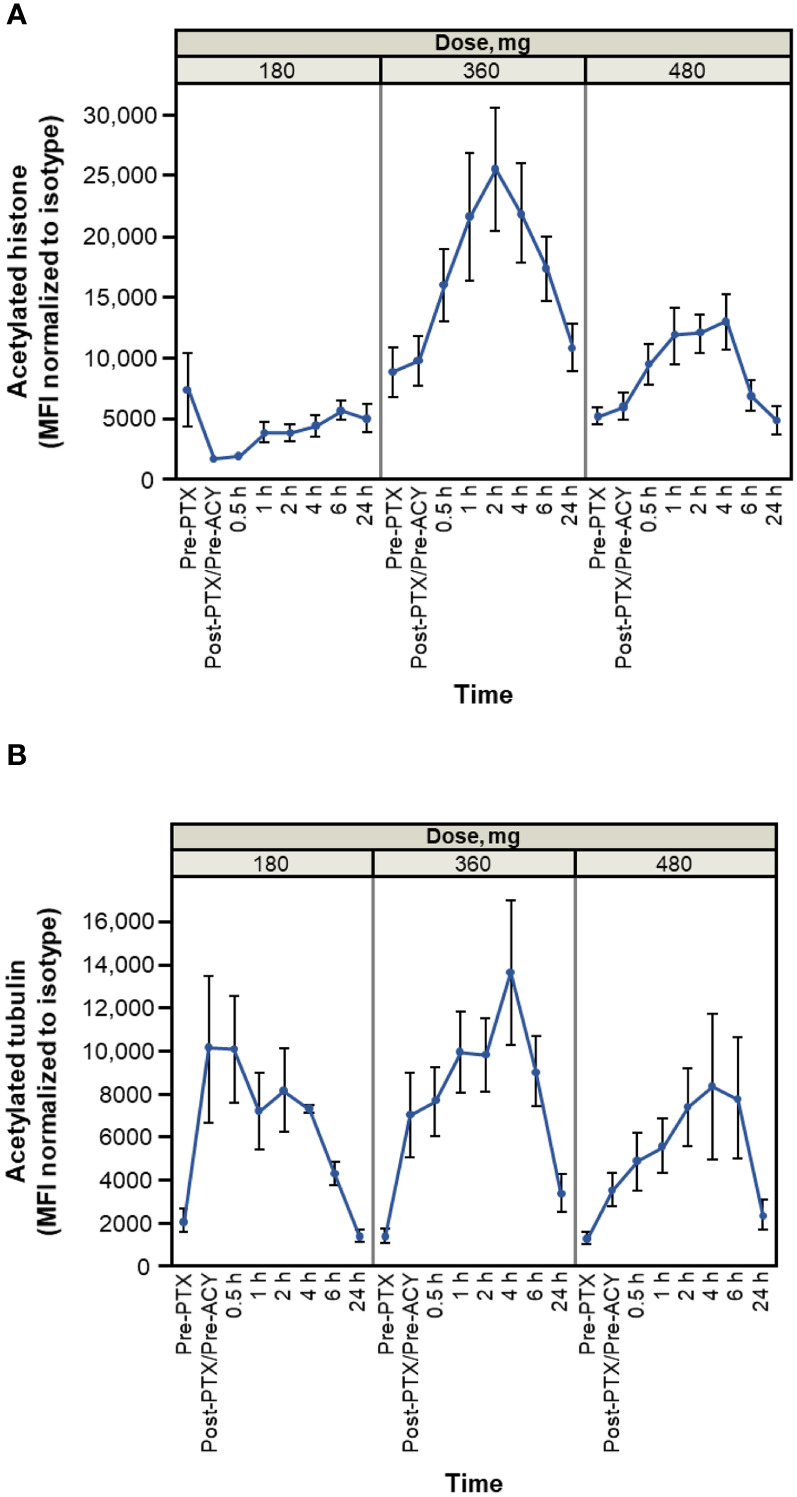
Histone **(A)** and tubulin **(B)** acetylation time course (average and SEM for patients treated at indicated levels on cycle 1 day 1). Dose levels indicated are 180, 360, and 480 mg of citarinostat orally once daily. Note: measurements are made on *n* = 3 at 180-mg level, *n* = 11 at 360-mg level, and *n* = 6 at 480-mg level. Cycle 1 day 15 measurements have similar profiles (data not shown). ACY, ACY-241 (citarinostat); MFI, mean fluorescence intensity; PTX, paclitaxel.

Results of pharmacodynamic changes from baseline in CD3^+^ PBMCs showed that increased acetylation levels of both histones and tubulin were detectable within 30 minutes of administration of citarinostat 360 or 480 mg and persisted for ≥ 6 hours (**Figure 3**). After paclitaxel administration and before citarinostat dosing, the level of tubulin acetylation was increased. The highest levels of acetylation of both histones and tubulin were noted at the 360-mg dose level. On the basis of these data as well as the safety results, the RP2D for citarinostat was determined to be 360 mg when used in combination with paclitaxel 80 mg/m^2^ qw 3/4.

## Discussion

In this phase Ib study, no unexpected toxicities or DLTs were observed with citarinostat in combination with paclitaxel in patients with heavily pretreated advanced solid tumors. As a result, the MTD was not reached, and the planned maximum administered dose showed no benefit over the next lower dose: at the 480- and 360-mg doses, 1 and 2 confirmed PRs, respectively, were observed. Citarinostat exposure doubled from the 180- to 360-mg doses but did not increase substantially with the 480-mg dose; with the 360- and 480-mg doses, exposure was generally similar from cycle 1 day 1 to day 15. One potential cause for the lack of exposure increase at the 480-mg dose could be saturable absorption, but this was not investigated further. Peak acetylation of histone and tubulin occurred at the 360-mg dose. On the basis of the safety, pharmacokinetic, and pharmacodynamic results, the RP2D of citarinostat was determined to be 360 mg once daily in combination with paclitaxel 80 mg/m^2^ qw 3/4. The tolerability of this dose was confirmed in an additional cohort of patients that included only those with disease that had previously progressed on a taxane. Overall, many patients were previously treated with taxanes, and that previous treatment did not preclude clinical benefit with this combination. In general, paclitaxel exposure decreased with increased doses of citarinostat. This finding is consistent with the observation that paclitaxel may be altered by inducers of cytochrome P450 (CYP) 2C8 and/or CYP3A4, and citarinostat is a moderate inducer of CYP1A2 and CYP3A4 (unpublished results).

Historically, the toxicity profile of pan-HDACi has been prohibitive and has limited their use in combination chemotherapy regimens. Several pan-HDACi approved for hematologic indications—including vorinostat, panobinostat, and romidepsin—have shown evidence of cardiac toxicity, specifically QT prolongation (18), which increases the risk of malignant cardiac arrhythmias and sudden cardiac death. As a selective HDAC6 inhibitor with less extensive inhibition of class I HDACs, citarinostat demonstrated an acceptable safety profile, with no reports of cardiac toxicity or QT prolongation. More hematologic toxicity was observed at the 360-mg than the 480-mg dose, but the pharmacokinetics does not suggest that this is a direct drug-mediated event. Patients in the 360-mg group more frequently had heavily pretreated cancers (such as ovarian cancer), which place patients at higher risk of hematologic toxicities, than patients in the 480-mg group (19). Pan-HDACi and chemotherapy combinations such as vorinostat, paclitaxel, and carboplatin have been associated with noted thrombocytopenia (15), but the only hematologic toxicities observed with citarinostat and paclitaxel were neutropenia, leukopenia, and anemia. Altered hematotoxicity with citarinostat and paclitaxel could be due to the HDAC6-specific inhibition of citarinostat or the citarinostat-induced changes to paclitaxel-based pharmacokinetics. Not surprisingly, most peripheral neuropathy events were likely due to paclitaxel treatment, which is known to be associated with this AE (20). The incidence of grade 3/4 peripheral neuropathy reported in this study (25%) was within the range of that reported previously with paclitaxel alone at various doses and schedules (2%–33%) (20), including 90 mg/m^2^ qw 3/4 (18%) (21). One patient discontinued treatment due to peripheral sensory neuropathy associated with paclitaxel. Two patients experienced ≥ 1 neuropathy-related TEAE associated with paclitaxel, leading to dose reduction. Collectively, these findings support the tolerability of the regimen.

The pharmacodynamic data revealed increased tubulin acetylation over time with citarinostat 360 and 480 mg as well as histone acetylation with citarinostat 360 mg (and less pronounced but noticeable histone acetylation with 480 mg). Tubulin acetylation levels increased in response to paclitaxel before citarinostat administration and subsequently increased further with citarinostat but only at the 360- or 480-mg dose level. Histone acetylation with citarinostat has been observed previously, albeit with higher drug concentrations than those required to increase tubulin acetylation (11). Although the sample numbers in our study were small, our findings suggest that citarinostat at the 360-mg dose may exhibit activity against both HDAC6 and class I HDACs and raise the possibility that a dose between 180 and 360 mg may be required to demonstrate the relative selectivity of the agent for HDAC6.

Interpretations of this phase Ib study of citarinostat plus paclitaxel are limited due to the small size of each cohort and lack of a comparator arm. The MTD of citarinostat was not reached, and an RP2D of 360 mg daily citarinostat in combination with paclitaxel 80 mg/m^2^ qw 3/4 was established. The safety profile and potential antitumor activity of citarinostat plus paclitaxel in patients with heavily pretreated advanced solid tumors reported in this study are encouraging; however, further studies are needed to evaluate whether there is clear evidence of efficacy with the addition of citarinostat or with this combination.

## Data Availability Statement

The original contributions presented in the study are included in the article/supplementary material. Further inquiries can be directed to the corresponding author.

## Ethics Statement

The studies involving human participants were reviewed and approved by the International Council for Harmonisation of Technical Requirements for Pharmaceuticals for Human Use and current Good Clinical Practice guidelines and the general ethical principles of the Declaration of Helsinki. This study was approved by the institutional review board at each participating site. The patients/participants provided their written informed consent to participate in this study.

## Author Contributions

MG: Conceptualization, Formal analysis, Investigation, Methodology, Project administration, Resources, Supervision, Writing – original draft, Writing – review and editing. GS: Conceptualization, Formal analysis, Investigation, Methodology, Project administration, Resources, Supervision, Writing – original draft, Writing – review and editing. JS: Conceptualization, Formal analysis, Investigation, Methodology, Project administration, Resources, Supervision, Writing – original draft, Writing – review and editing. DJ: Conceptualization, Formal analysis, Investigation, Methodology, Project administration, Resources, Supervision, Writing – original draft, Writing – review and editing. BL: Conceptualization, Formal analysis, Methodology, Project administration, Resources, Supervision, Validation, Writing – original draft, Writing – review and editing. AZ: Data curation, Formal analysis, Validation, Writing – original draft, Writing – review and editing. JC: Conceptualization, Data curation, Formal analysis, Methodology, Resources, Supervision, Validation, Writing – original draft, Writing – review and editing. YLB: Conceptualization, Data curation, Formal analysis, Methodology, Project administration, Resources, Supervision, Validation, Writing – original draft, Writing – review and editing. CD: Conceptualization, Data curation, Formal analysis, Methodology, Resources, Supervision, Validation, Writing – original draft, Writing – review and editing. RH: Conceptualization, Formal analysis, Investigation, Methodology, Project administration, Resources, Supervision, Writing – original draft, Writing – review and editing. All authors contributed to the article and approved the submitted version.

## Funding

Funding for this research was provided by Celgene, a Bristol-Myers Squibb Company, Summit, NJ, and an NCI Cancer Center Support grant (#P30 CA054174) to the Institute for Drug Development, Cancer Therapy and Research Center at The University of Texas Health Science Center at San Antonio, San Antonio, TX.

## Conflict of Interest

MG: employment, Arizona Center for Cancer Care; stock, Medelis, Care Mission USA; honoraria, Deciphera; consulting/advisory role, Deciphera, RedHill Biopharma, Salarius; research funding, AbbVie, Acetylon, Celgene, a Bristol-Myers Squibb Company, Celldex, Corcept, Calithera, Deciphera, Eisai, Endocyte, Eli Lilly, Five Prime, Genentech, Plexxikon, Pfizer, MedImmune, Merck, OncoMed. GS: research funding, Eli Lilly, Merck KGaA/EMD Serono, Merck, Sierra Oncology; consulting/advisory role, Almac, Angiex, Astex, Bayer, Bicycle Therapeutics, Cybrexa Therapeutics, Daiichi Sankyo, Eli Lilly, Fusion Pharmaceuticals, G1 Therapeutics, Ipsen, Merck KGaA/EMD Serono, Pfizer, Roche, Sierra Oncology. DJ: consulting/advisory role, Eisai, EMD Serono, Novartis. BL, YLB, and CD: employment, Bristol Myers Squibb; stock, Bristol Myers Squibb. AZ: employment, Celgene, a Bristol-Myers Squibb Company; stock, Bristol Myers Squibb. JC: employment, Bristol Myers Squibb; stock, Bristol Myers Squibb; research funding, Celgene, a Bristol-Myers Squibb Company; travel, Celgene, a Bristol-Myers Squibb Company. RH: consulting/advisory, Amgen, GlaxoSmithKline; research funding, Abbisko, AbbVie, Actuate, Alkermes, Amgen, AstraZeneca, Bayer, Bristol Myers Squibb, Boston Biomedical, Calithera, Celgene, a Bristol-Myers Squibb Company, FujiFilm, Genmab, GlaxoSmithKline, Infinity, InhibRx, Merck, Mersana, Meryx, Nektar, Pfizer, Puma, RAPT Therapeutics, Regeneron, Rgenix, Sanofi, Seattle Genetics, Sutro, Takeda, Xencor. The authors declare that this study received funding from Celgene Research S.L.U. The funder was involved in the study design, collection, analysis, interpretation of data, and funded the writing of this article.

The remaining author declares that the research was conducted in the absence of any commercial or financial relationships that could be construed as a potential conflict of interest.

## Publisher’s Note

All claims expressed in this article are solely those of the authors and do not necessarily represent those of their affiliated organizations, or those of the publisher, the editors and the reviewers. Any product that may be evaluated in this article, or claim that may be made by its manufacturer, is not guaranteed or endorsed by the publisher.
